# Prevalence of macrolide and fluoroquinolone resistance-mediating mutations in *Mycoplasma genitalium* in five cities in Russia and Estonia

**DOI:** 10.1371/journal.pone.0175763

**Published:** 2017-04-13

**Authors:** Elena Shipitsyna, Tatiana Rumyantseva, Daniel Golparian, Guzel Khayrullina, Amaya C. Lagos, Inna Edelstein, Kai Joers, Jörgen S. Jensen, Alevtina Savicheva, Natalia Rudneva, Larisa Sukhanova, Roman Kozlov, Alexander Guschin, Magnus Unemo

**Affiliations:** 1 Laboratory of Microbiology, D.O. Ott Research Institute of Obstetrics, Gynaecology and Reproductology, St. Petersburg, Russia; 2 WHO Collaborating Centre for Gonorrhoea and Other STIs, Department of Laboratory Medicine, Faculty of Medicine and Health, Örebro University, Örebro, Sweden; 3 Laboratory of Molecular Diagnostics, Central Research Institute of Epidemiology, Moscow, Russia; 4 Institute of Antimicrobial Chemotherapy, Smolensk State Medical University, Smolensk, Russia; 5 United Laboratories, Tartu University Hospital, Tartu, Estonia; 6 Department of Microbiology and Infection Control, Sexually Transmitted Infections, Research and Development, Statens Serum Institut, Copenhagen, Denmark; 7 Tula Regional Clinic of Dermato-Venereology of the Ministry of Health of the Tula Region, Tula, Russia; The University of Melbourne, AUSTRALIA

## Abstract

**Background and objective:**

Resistance in the sexually transmitted bacterium *Mycoplasma genitalium* to all recommended therapeutic antimicrobials have rapidly emerged. However, to date, internationally reported resistance surveillance data for *M*. *genitalium* strains circulating in Eastern Europe are entirely lacking. The aim of this study was to estimate the prevalence of macrolide and fluoroquinolone resistance-associated mutations in *M*. *genitalium* in four cities in Russia and one in Estonia, 2013–2016.

**Materials and methods:**

Consecutive urogenital samples found positive for *M*. *genitalium* during diagnostic testing were retrospectively analyzed for resistance-associated mutations in the 23S rRNA and *parC* genes using pyrosequencing and conventional Sanger sequencing, respectively.

**Results:**

In total, 867 *M*. *genitalium* positive samples from 2013–2016 were analyzed. Macrolide resistance-associated mutations were detected in 4.6% of the samples from Russia (0.7–6.8% in different cities) and in 10% of the samples from Estonia. The mutations A2059G and A2058G were highly predominating in both Russia and Estonia, accounting together for 90.9% of the cases positive for nucleotide substitutions in the 23S rRNA gene. The rates of possible fluoroquinolone resistance-associated mutations were 6.2% in Russia (2.5–7.6% in different cities) and 5% in Estonia. The mutations S83I and S83N were the most frequent ones in Russia (24.4% each), whereas D87N highly predominated in Estonia (83.3% of all fluoroquinolone resistance-associated mutations). Approximately 1% of the samples in both countries harbored both macrolide and possible fluoroquinolone resistance-associated mutations, with A2058G and S83I being the most frequent combination (37.5%).

**Conclusions:**

The prevalence of macrolide and fluoroquinolone resistance-associated mutations in *M*. *genitalium* was 4.6% and 6.2%, respectively, in Russia, and 10% and 5%, respectively, in Estonia. Despite the relatively low rates of macrolide and fluoroquinolone resistance in these countries, antimicrobial resistance surveillance and testing for resistance-associated mutations in *M*. *genitalium* positive cases would be valuable.

## Introduction

*Mycoplasma genitalium* frequently causes urethritis in men, and urethritis and cervicitis in women [1]. *M*. *genitalium* is also associated with an increased risk of pelvic inflammatory disease, preterm birth, spontaneous abortion and tubal factor infertility [2]. In men with symptomatic non-gonococcal urethritis (NGU), *M*. *genitalium* has been detected in 15% to 25% of cases [1].

The recommended syndromic first-line treatment for male NGU in the 2016 European guideline is doxycycline 100 mg twice daily or 200 mg once daily orally for seven days. Second-line regimen is azithromycin 500 mg single dose, then 250 mg daily for 4 days, or azithromycin 1 g single dose orally [3]. In the 2016 European guideline on *M*. *genitalium* infections, the extended azithromycin regimen is the first-line treatment when macrolide resistance status is unknown. Patients with *M*. *genitalium* macrolide-resistant strains should be treated with moxifloxacin 400 mg once daily for 7(-10) days [4],[5]. Early randomized controlled clinical trials demonstrated that azithromycin was superior to doxycycline for the treatment of *M*. *genitalium*-associated urethritis. However, during recent years the efficacy of azithromycin 1 g single dose has steadily declined and is now approaching 60% [1],[4],[5],[6]. The extended regimen of azithromycin, 500 mg day one followed by 250 mg days 2–5, is more efficacious than 1 g single dose treatment [1],[4],[5]. Nevertheless, also the extended azithromycin regimen fails to treat azithromycin-resistant *M*. *genitalium* infections [7]. The declining azithromycin efficacy is caused by increasing prevalence of macrolide resistance, primarily mediated by nucleotide substitutions at positions A2058 or A2059 (*Escherichia coli* numbering) in region V of the 23S rRNA gene [8].

The recommended second-line treatment for *M*. *genitalium* infections, the fluoroquinolone moxifloxacin [4], was initially highly efficacious [1],[5]. However, during the most recent years a declining eradication rate has been documented, particularly in the Asia-Pacific region, with treatment failures in up to 30% of cases [9],[10],[11]. The resistance to moxifloxacin in *M*. *genitalium* is mediated by mutations in the quinolone resistance determining region (QRDR) of the *parC* gene, primarily at amino acid positions S83 and D87 (*M*. *genitalium* numbering) [12],[13].

Test of cure more than 21 days after treatment is recommended in the European guideline [4] and surveillance of *M*. *genitalium* antimicrobial resistance is crucial. To date, internationally reported resistance surveillance data for *M*. *genitalium* strains circulating in Eastern Europe are entirely lacking.

This study estimated the prevalence of macrolide and fluoroquinolone resistance in *M*. *genitalium* in four cities in Russia and one in Estonia.

## Materials and methods

### Biological samples

The study was performed as a retrospective analysis of urogenital samples found positive for *M*. *genitalium* during diagnostic testing, mainly using the AmpliSеns N.gonorrhoeae/C.trachomatis/M.genitalium/T.vaginalis-MULTIPRIME-FRT PCR assay or AmpliSens Mycoplasma genitalium-FRT PCR assay (InterLabService, Moscow, Russia) or Anyplex II STI-7 PCR assay (Seegene, Seoul, Korea), at five participating centres: 1) Laboratory of Molecular Diagnostics, Central Research Institute of Epidemiology, Moscow, Russia; 2) Laboratory of Microbiology, D.O. Ott Research Institute of Obstetrics, Gynaecology and Reproductology, St Petersburg, Russia; 3) Institute of Antimicrobial Chemotherapy, Smolensk State Medical University, Smolensk, Russia; 4) Tula Regional Clinic of Dermato-Venereology of the Ministry of Health of the Tula Region, Tula, Russia; and 5) United Laboratories, Tartu University Hospital, Tartu, Estonia. The samples were collected consecutively from outpatients attending gynaecological, urological and dermatovenereological clinics due to urogenital symptoms, partner notification or high-risk sexual behavior predominantly in 2013–2016. One specimen per male patient (mostly urethral swab samples) or female patient (mostly cervical swab samples) on a specific date was included. All examined specimens were sampled and stored as part of the routine diagnostics (standard care), and no patient identification information was available in the study. Accordingly, no ethical approval was required for the study.

### DNA isolation

Genomic DNA was isolated from 200 μl of the primary sample using NucliSens easyMAG (bioMérieux, France) or MagNA Pure (Roche, Germany). The DNA preparations were stored at -70°C prior to antimicrobial resistance testing.

### Detection of resistance-associated mutations

Resistance-associated mutations in the 23S rRNA and *parC* genes were identified using pyrosequencing and conventional Sanger sequencing, respectively, as described previously [8],[12].

### Statistics

For testing differences in country- and city-specific rates of mutations, chi-square statistics were computed using IBM SPSS Statistics software (IBM).

## Results

### *Mycoplasma genitalium* positive samples

In total, 867 *M*. *genitalium* positive urogenital samples (408 (47%) from females, 275 (32%) from males, and 184 (21%) unspecified) were analyzed. In Moscow, 406 samples were obtained in 2014. In St Petersburg, 59 samples were collected: 30 in 2013, 26 in 2014, and three in 2015. In Smolensk, 127 samples were obtained: 19 in 2009–2012, 24 in 2013, 28 in 2014, and 56 in 2015. In Tula, 142 samples were obtained in 2015. In Tartu, 133 samples were collected: 100 samples in 2014 and 33 samples in 2016. The targeted region of the 23S rRNA gene and the *parC* gene could be PCR amplified and sequenced in 829 (95.6%) samples and 783 (90.3%) samples, respectively. Most samples that could not be PCR amplified in the antimicrobial resistance testing were low positive in the initial diagnostic PCR. Both target sequences were successfully sequenced in 766 (88.4%) samples.

### Macrolide resistance

Macrolide resistance-associated 23S rRNA gene mutations were detected in 4.6% of the samples from Russia and in 10% of the samples from Estonia (*p* = 0.018) (Table 1).

**Table 1:** Prevalence of macrolide resistance-associated 23S rRNA gene mutations in *Mycoplasma genitalium* positive samples in four cities in Russia and one city in Estonia.

Mutation^a^	Frequency, % (No of samples containing mutation(s)/No of successfully sequenced samples)						
	Russia					Estonia	Total
	Moscow	St Petersburg	Smolensk	Tula	Total	Tartu	
A2059G	3.6 (14/391)	6.8 (4/59)	1.6 (2/127)		2.8 (20/719)	6.4 (7/110)	3.3 (27/829)
A2058G	1.3 (5/391)		3.1 (4/127)	0.7 (1/142)	1.4 (10/719)	2.7 (3/110)	1.6 (13/829)
A2058C	0.3 (1/391)				0.1 (1/719)		0.1 (1/829)
A2058T	0.3 (1/391)				0.1 (1/719)		0.1 (1/829)
A2062G	0.3 (1/391)				0.1 (1/719)		0.1 (1/829)
C2055G						0.9 (1/110)	0.1 (1/829)
Total	5.6 (22/391)	6.8 (4/59)	4.7 (6/127)	0.7 (1/142)	4.6 (33/719)	10 (11/110)	5.3 (44/829)
		*In 2013*: *6*.*7 (2/30); in 2014*: *7*.*7 (2/26); in 2015*: *0 (0/3)*	*In 2009–2012*: *5*.*3 (1/19); in 2013*: *4*.*2 (1/24); in 2014*: *10*.*7 (3/28); in 2015*: *1*.*8 (1/56)*			*In 2014*: *7*.*1 (6/84); in 2016*: *19*.*2 (5/26)*	

^a^The nucleotide positions in the 23S rRNA gene are given according to *Escherichia coli* numbering.

In Russia, the highest frequency of macrolide resistance-associated mutations was revealed in St Petersburg (6.8%), followed by Moscow (5.6%), Smolensk (4.7%), and Tula (0.7%) (*p* = 0.089). The mutations A2059G (n = 27) and A2058G (n = 13) were highly predominating in both Russia and Estonia, accounting together for 90.9% (40/44) of the cases positive for nucleotide substitutions in the 23S rRNA gene. The remaining mutations (A2058C, A2058T, A2062G, and C2055G) were only found in one sample each (Table 1).

### Fluoroquinolone resistance

The rates of possible fluoroquinolone resistance-associated mutations (mutations in QRDR of *parC*) were similar in Russia (6.2%) and Estonia (5%) (*p* = 0.599) (Table 2).

**Table 2:** Prevalence of possible fluoroquinolone resistance-associated *parC* mutations (mutations in QRDR of *parC*) in *Mycoplasma genitalium* positive samples in four cities in Russia and one city in Estonia.

Mutation(s)^a^	Frequency, % (No of samples containing mutation(s)/No of successfully sequenced samples)						
	Russia					Estonia	Total
	Moscow	St Petersburg	Smolensk	Tula	Total	Tartu	
D87N^b^	1.0 (4/381)		0.9 (1/108)		0.8 (5/662)	4.1 (5/121)	1.3 (10/783)
S83I^b^	2.4 (9/381)		0.9 (1/108)		1.5 (10/662)		1.3 (10/783)
S83N^c^	2.4 (9/381)		0.9 (1/108)		1.8 (10/662)		1.3 (10/783)
D87Y^b^	0.5 (2/381)		0.9 (1/108)	0.8 (1/122)	0.6 (4/662)		0.5 (4/783)
S83R^b^	0.5 (2/381)				0.3 (2/662)		0.3 (2/783)
S83V			0.9 (1/108)		0.2 (1/662)		0.1 (1/783)
S84G	0.3 (1/381)				0.2 (1/662)		0.1 (1/783)
S84H	0.3 (1/381)				0.2 (1/662)		0.1 (1/783)
S84I						0.8 (1/121)	0.1 (1/783)
S84P	0.3 (1/381)				0.2 (1/662)		0.1 (1/783)
S84R		2.0 (1/51)			0.2 (1/662)		0.1 (1/783)
D87R				0.8 (1/122)	0.2 (1/662)		0.1 (1/783)
D87G			0.9 (1/108)		0.2 (1/662)		0.1 (1/783)
I90N		2.0 (1/51)			0.2 (1/662)		0.1 (1/783)
S83I and D87R				0.8 (1/122)	0.2 (1/662)		0.1 (1/783)
S84Y and D87N			0.9 (1/108)		0.2 (1/662)		0.1 (1/783)
Total	7.6 (29/381)	3.9 (2/51)	6.5 (7/108)	2.5 (3/122)	6.2 (41/662)	5 (6/121)	6 (47/783)
		*In 2013*: *8 (2/25); in 2014*: *0 (0/24); in 2015*: *0 (0/2)*	*In 2009–2012*: *6*.*3 (1/16); in 2013*: *8*.*3 (2/24); in 2014*: *12*.*5 (3/24); in 2015*: *2*.*3 (1/44)*			*In 2014*: *5*.*1 (5/98); in 2016*: *4*.*3 (1/23)*	

^a^The amino acid positions in ParC are given according to *M*. *genitalium* numbering.

^b^Mutations where *in vitro* MIC determination has demonstrated significantly elevated MICs of moxifloxacin.

^c^One strain evaluated had a moxifloxacin susceptible phenotype (Jensen et al, unpublished).

Among the Russian centres, the highest rate of *parC* QRDR mutations was found in Moscow (7.6%), followed by Smolensk (6.5%), St Petersburg (3.9%), and Tula (2.5%) (*p* = 0.194). The mutations D87N (n = 10), S83I (n = 10), S83N (n = 10), D87Y (n = 4), and S83R (n = 2) were the most frequent ones, accounting for 36 (76.6%) of 47 samples with amino acid alterations in ParC. In Estonia, the mutation D87N predominated (83.3%). The additional amino acid changes (S83V, S84G, S84H, S84I, S84P, S84R, D87R, D87G, and I90N) were only detected in one sample each. In two samples (both from Russia), two amino acid alterations in ParC (S83I+D87R and S84Y+D87N) were present (Table 2).

### Multidrug resistance

Eight samples, seven from Russia (1.1%) and one from Estonia (0.9%), harboured both macrolide and possible fluoroquinolone resistance-associated mutations (Table 3).

**Table 3:** Prevalence of *Mycoplasma genitalium* positive samples with multidrug resistance (both 23S rRNA gene and ParC mutations) in four cities in Russia and one city in Estonia.

Mutation(s)^a^	Frequency, % (No of samples containing mutation(s)/No of successfully sequenced samples)						
	Russia					Estonia	Total
	Moscow	St Petersburg	Smolensk	Tula	Total	Tartu	
A2058G and S83I	0.5 (2/378)		0.9 (1/108)		0.5 (3/659)		0.4 (3/766)
A2058C and S83N	0.3 (1/378)				0.2 (1/659)		0.1 (1/766)
A2058G and S84G	0.3 (1/378)				0.2 (1/659)		0.1 (1/766)
A2058G and D87G			0.9 (1/108)		0.2 (1/659)		0.1 (1/766)
A2058G and D87N						0.9 (1/107)	0.1 (1/766)
A2059G and S83I	0.3 (1/378)				0.2 (1/659)		0.1 (1/766)
Total	1.3 (5/378)	0 (0/51)	1.9 (2/108)	(0/122)	1.1 (7/659)	0.9 (1/107)	1 (8/766)
			*In 2009–2012*: *6*.*3 (1/16); in 2013*: *0 (0/24); in 2014*: *4*.*2 (1/24); in 2015*: *0 (0/44)*			*In 2014*: *1*.*2 (1/84); in 2016*: *0 (0/23)*	

^a^The nucleotide positions in the 23S rRNA gene are given according to *Escherichia coli* numbering, and the amino acid positions in ParC are given according to *M*. *genitalium* numbering.

A2058G and S83I was the most frequent combination of mutations (37.5% (3/8) of cases with multidrug resistance), whereas the other combinations (A2058C+S83N, A2058G+S84G, A2058G+D87G, A2058G+D87N, and A2059G+S83I) were present in only one sample each.

## Discussion

This study is the first large-scale surveillance of the prevalence of macrolide and fluoroquinolone resistance-associated mutations in *M*. *genitalium* in Eastern Europe. Previously, only one minor study (including 47 *M*. *genitalium* positive men in 2006–2008) from Eastern Europe involving *M*. *genitalium* antimicrobial resistance has been internationally published [14].

In recent years, the rates of *M*. *genitalium* macrolide resistance-associated mutations have been shown to vary significantly internationally, i.e. 10% in South Africa [15], 14–17% in France [16],[17] 30% in the Netherlands [18], 38% in Denmark [19], 41% in the UK [20], 36–43% in Australia [11],[21], 29–47% in Japan [22],[23], 48% in the USA [24], 53% in Germany [25], and 100% in Greenland [26]. In the present study, the prevalence of macrolide resistance-associated mutations was shown to be relatively low in Russia (4.6%), ranging from 0.7% in Tula (Central Federal District) to 6.8% in St Petersburg (Northwestern Federal District), and Estonia (10%). The resistance mutations at positions A2058 and A2059 [5],[8] were overwhelmingly predominant. However, also a single A2062G mutation in a sample from Moscow and a single C2055G mutation in a sample from Tartu were found. The mutation A2062G has previously been described in a *M*. *genitalium* treatment failure with josamycin in Russia [14], and an A2062T mutation has also been described in one *M*. *genitalium* positive pre-treatment sample in France [27]. Furthermore, A2062G and A2062T mutations have been selected *in vitro* with josamycin in *M*. *hominis* and *M*. *pneumoniae* and shown to cause high-level josamycin resistance [28],[29]. To our knowledge, the remaining C2055G mutation has not previously been described in mycoplasmas and its association with resistance to macrolides remains unknown.

The relatively low rate of macrolide resistance-associated mutations in Russia and Estonia, and the significant difference in macrolide resistance rates in these countries, can be attributed to a number of factors. First, testing for *M*. *genitalium* is not introduced everywhere, which may be particularly the case in Russia, and therefore many *M*. *genitalium* infections, symptomatic and particularly asymptomatic ones, are not diagnosed and treated. In Russia, in addition, some of the *M*. *genitalium* nucleic acid amplification tests have suboptimal sensitivity, which results in missing cases with low *M*. *genitalium* loads [30]. Furthermore, screening for *Chlamydia trachomatis*, with subsequent treatment using azithromycin, is also less frequent in Russia compared to many European Union countries. Finally, in Russia the recommended first-line antimicrobials for *M*. *genitalium* infections are josamycin (500 mg three times daily, 10 days) or doxycycline (100 mg twice a day, 10 days) [31]. In Estonia, the recommended first-line antimicrobials are azithromycin (500 mg first day, then 250 mg daily for 4 days) or as alternative treatment moxifloxacin 400 mg daily for 7–10 days. The data on activity of josamycin against *M*. *genitalium* and the resistance selection with josamycin are very limited. Its activity *in vitro* was shown to be slightly lower than that of azithromycin [32]. It was found that macrolide resistance mutations resulting in treatment failures can be rapidly selected during treatment with josamycin [14], with a mutation selection rate of 6.5%, which is comparable with that of the extended course of azithromycin (0–6.5%), yet lower than that of 1 g single dose azithromycin (10%) [7],[33]. It is unknown how frequently doxycycline is administered for *M*. *genitalium* infections in Russia (can vary substantially in different regions), but using this drug may contribute to the decreased rate of macrolide resistance as a proportion (~30%) of patients get cured with the recommend doxycycline regimen. Nevertheless, the macrolide resistance selection pressure is still considerable in Russia, particularly because josamycin and azithromycin (1 g single oral dose) are widely used as first-line treatments for *C*. *trachomatis* infections.

Data regarding the prevalence of possible fluoroquinolone resistance-associated mutations in Europe remain very limited. In mostly minor recent studies from United Kingdom, France and Germany, a ParC QRDR alteration was found in 5% (1 of 22 samples) [20], 6% (12/200) [17] and 10% (2/19) [25] of samples, respectively. In contrast, in a study from Australia, 15% of specimens collected between 2008 and 2011 carried mutations associated with fluoroquinolone resistance [21]. A report from Japan found *parC* QRDR mutations in 33% of specimens collected between 2011 and 2013, with a dramatic increase from 20% in 2011 to 47% in 2013 [22]. However, a majority of this increase was due to the S83N mutation that might not result in significantly elevated MICs of moxifloxacin (Jensen et al, unpublished). In our study, the rates of *parC* QRDR mutations were 6.2% in Russia, ranging from 2.5% in Tula to 7.6% in Moscow, and 5% in Estonia. The predominance of the mutations S83I, S83N, D87N and D87Y in our study is consistent with previous studies [11],[13],[21],[22]. In addition to nonsynonymous mutations at S83 and D87 (80 and 84 in *E*. *coli*), which are known to be associated with fluoroquinolone resistance in *M*. *genitalium* and other closely related organisms [11],[13],[21],[22],[34], we found several amino acid changes at position 84 (81 in *E*. *coli*) (S84G, S84H, S84I, S84R, S84Y) and one mutation at position 90 (87 in *E*. *coli*) (I90N), which have not been previously reported or associated with fluoroquinolone resistance, to our knowledge, in mycoplasmas. However, also a ParC S84P mutation was found in one sample from Moscow and this mutation has been previously shown to cause resistance to fluoroquinolones in a clinical *M*. *hominis* isolate [35]. The recommended second-line treatment for *M*. *genitalium* infections in Russia is ofloxacin (400 mg twice a day, 10 days) [31], although other fluoroquinolones, including moxifloxacin and levofloxacin, can also be used. Given the suboptimal activity of ofloxacin and levofloxacin against *M*. *genitalium* and the frequent use of these fluoroquinolones in the treatment of other gynaecological and urological infections (which exerts pressure to select fluoroquinolone resistance in *M*. *genitalium*), the relatively low rate of fluoroquinolone resistance mutations in Russia was slightly unexpected. However, the possible resistance to fluoroquinolones was higher than the resistance to macrolides, which is rare internationally.

In the present study, seven samples in Russia and one sample in Estonia harbored both macrolide and possible fluoroquinolone resistance-associated mutations, accounting for about 1% of samples in both countries. Multidrug resistance in *M*. *genitalium*, with a prevalence of up to 25%, has recently been reported in studies from Australia and Japan [10],[11],[21],[22],[23]. This is of great concern because there is only one option for third-line treatment, i.e. pristinamycin (1 g four times daily for 10 days) [4], which is not 100% effective. Novel antimicrobials for treatment of *M*. *genitalium* infections are crucial and dual antimicrobial therapy has to be considered. Some new antimicrobials such as solithromycin, lefamulin, and zoliflodacin deserve further attention for treatment of *M*. *genitalium* infections [5].

The main limitations of the present study included that no information was available whether a sample was submitted before or after exposure to any antimicrobial treatment. In many cases, it was not either possible to reliably identify multiple *M*. *genitalium* positive samples from the identical individuals, because no unique patient-specific numbers are used in Russia and, in addition, in some clinics patients can submit their samples anonymously. Consequently, there might have been a few cases of treatment failures in the examined material, creating a limited bias in the prevalence of mutations. It was also impossible to compare resistance rates in different years, because nearly 90% of the samples were obtained in 2014 (n = 560; 64.6%) and 2015 (n = 201; 23.2%), and also because the samples obtained in different years were not representative of all centres.

## Conclusions

The prevalence of macrolide and fluoroquinolone resistance-associated mutations in *M*. *genitalium* in Russia was 4.6% (ranging from 0.7 to 6.8% in different regions) and 6.2% (ranging from 2.5 to 7.6% in different regions), respectively, and in Estonia 10% and 5%, respectively. Despite the relatively low rates of macrolide and fluoroquinolone resistance in these countries, antimicrobial resistance surveillance would be valuable, and *M*. *genitalium* positive cases should ideally be screened for resistance-associated mutations before initiation of treatment. Unfortunately, the importance of a number of mutations in the 23S rRNA gene and particularly the *parC* gene remains unknown. This calls for prioritizing culture of samples with such mutations in order to associate mutations with phenotypic resistance and treatment outcome. In *M*. *genitalium* strains that are currently circulating and have been cultured, the specific *parC* QRDR mutations appear to explain the phenotypic resistance to moxifloxacin. However, an impact of *gyrA* QRDR mutations to further increase the MICs of moxifloxacin in *M*. *genitalium* strains spreading in the future cannot be excluded. Ultimately, novel antimicrobials for treatment of *M*. *genitalium* infections are crucial and dual antimicrobial therapy has to be considered.
